# Effect of Different Gate Lengths on Polarization Coulomb Field Scattering Potential in AlGaN/GaN Heterostructure Field-Effect Transistors

**DOI:** 10.1038/s41598-018-27357-6

**Published:** 2018-06-13

**Authors:** Peng Cui, Jianghui Mo, Chen Fu, Yuanjie Lv, Huan Liu, Aijie Cheng, Chongbiao Luan, Yang Zhou, Gang Dai, Zhaojun Lin

**Affiliations:** 1 School of Microelectronics, Shandong University, Jinan, 250100 China; 2 National Key Laboratory of Application Specific Integrated Circuit (ASIC), Hebei Semiconductor Research Institute, Shijiazhuang, 050051 China; 3 School of Mathematics, Shandong University, Jinan, 250100 China; 4 Key Laboratory of Pulsed Power, Institute of Fluid Physics, CAEP, Mianyang, 621999 China; 5 Microsystem and Terahertz Research Center, China Academy of Engineering Physics, Chengdu, 610200 China

## Abstract

The AlGaN/GaN heterostructure field-effect transistors with different gate lengths were fabricated. Based on the chosen of the Hamiltonian of the system and the additional polarization charges, two methods to calculate PCF scattering by the scattering theory were presented. By comparing the measured and calculated source-drain resistances, the effect of the different gate lengths on the PCF scattering potential was confirmed.

## Introduction

AlGaN/GaN heterostructure field-effect transistors (HFETs) have emerged as excellent devices for high-frequency and high-power applications^1–14^, owing to their superior properties such as high electron mobility, high saturation electron velocity, and large critical breakdown field^15–25^. Polarization Coulomb field (PCF) scattering, stemming from the non-uniform distribution of the strain in the AlGaN barrier layer, can affect the electron mobility, parasitic source access resistance, transconductance, and device linearity in AlGaN/GaN HFETs^26–40^. Based on the perturbation theory, the theoretical model of PCF scattering has been established^31^, which makes it possible to quantitative study of PCF scattering^35–40^. The Hamiltonian of electrons in the source-drain channel can be split into two parts $\hat{H}={\hat{H}}_{0}+\hat{H}^{\prime}$, where ${\hat{H}}_{0}$ is known as the Hamiltonian of the unperturbed system, and $\hat{H}^{\prime}$ is called the perturbation. For PCF scattering, $\hat{H}^{\prime}$ originates from the effect of additional polarization charges on the channel electrons, and is referred as the PCF scattering potential. The polarization charges underneath the gate region can be altered by the gate bias^41^, leading to the polarization charges at the AlGaN/GaN interface uneven. The non-uniform distribution of polarization charges can generate the PCF scattering potential. The gate length is relevant with the non-uniform distribution of polarization charges^26,30,35,38^. Hence, the different gate lengths can affect the PCF scattering potential. If the PCF scattering potential is large enough, the accuracy of the PCF scattering model based on the perturbation theory may be challenged. Therefore, exploring the effect of different gate lengths on PCF scattering potential is necessary.

In this paper, the AlGaN/GaN HFETs with different gate lengths were fabricated. By comparing the measured and calculated source-drain resistances, the influence of different gate lengths on PCF scattering potential was explored.

## Results and Discussion

**Figure 1:**
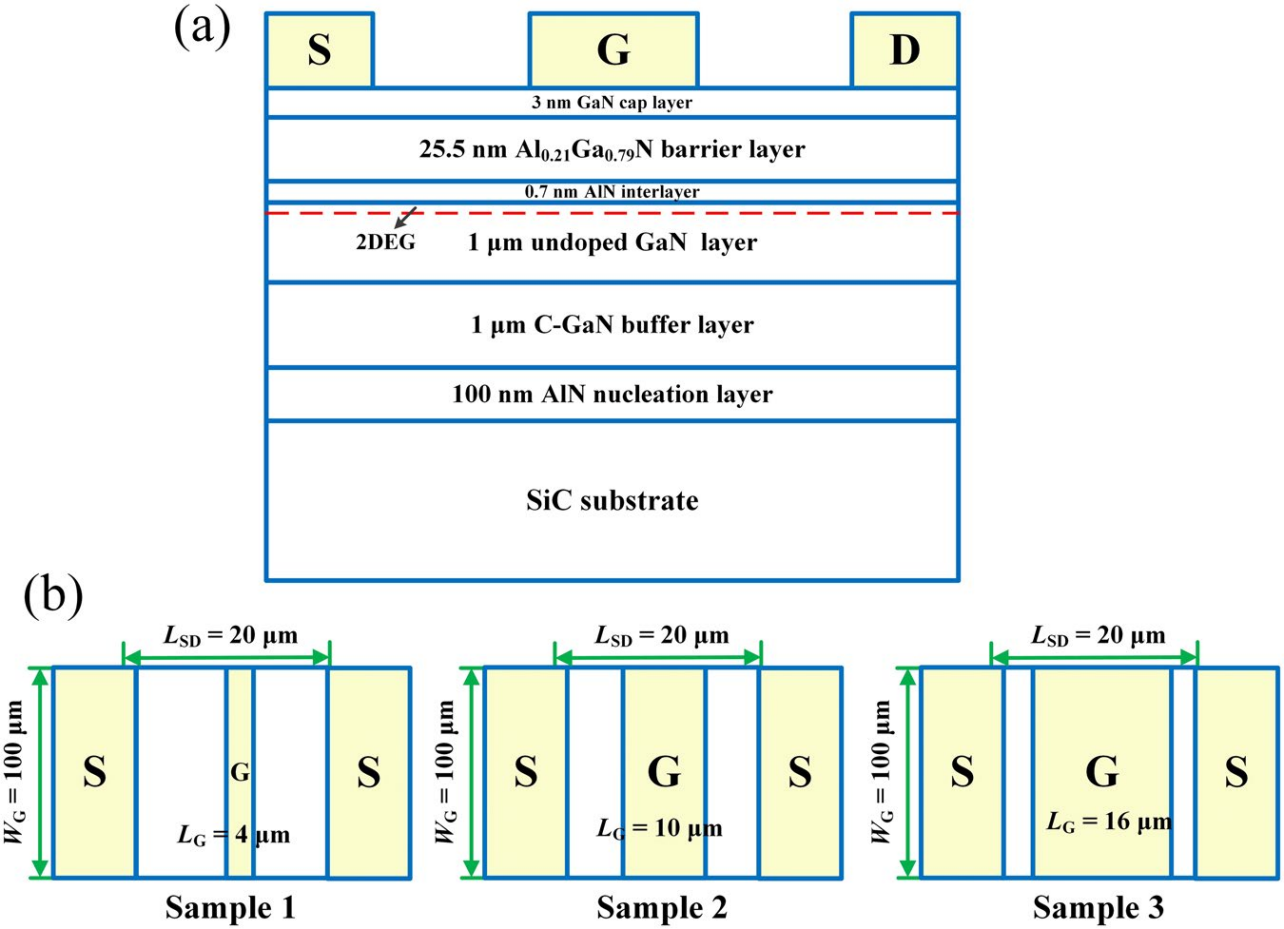
(**a**) Cross-sectional view of the AlGaN/GaN HFETs. (**b**) Top view of the three samples.

**Figure 2:**
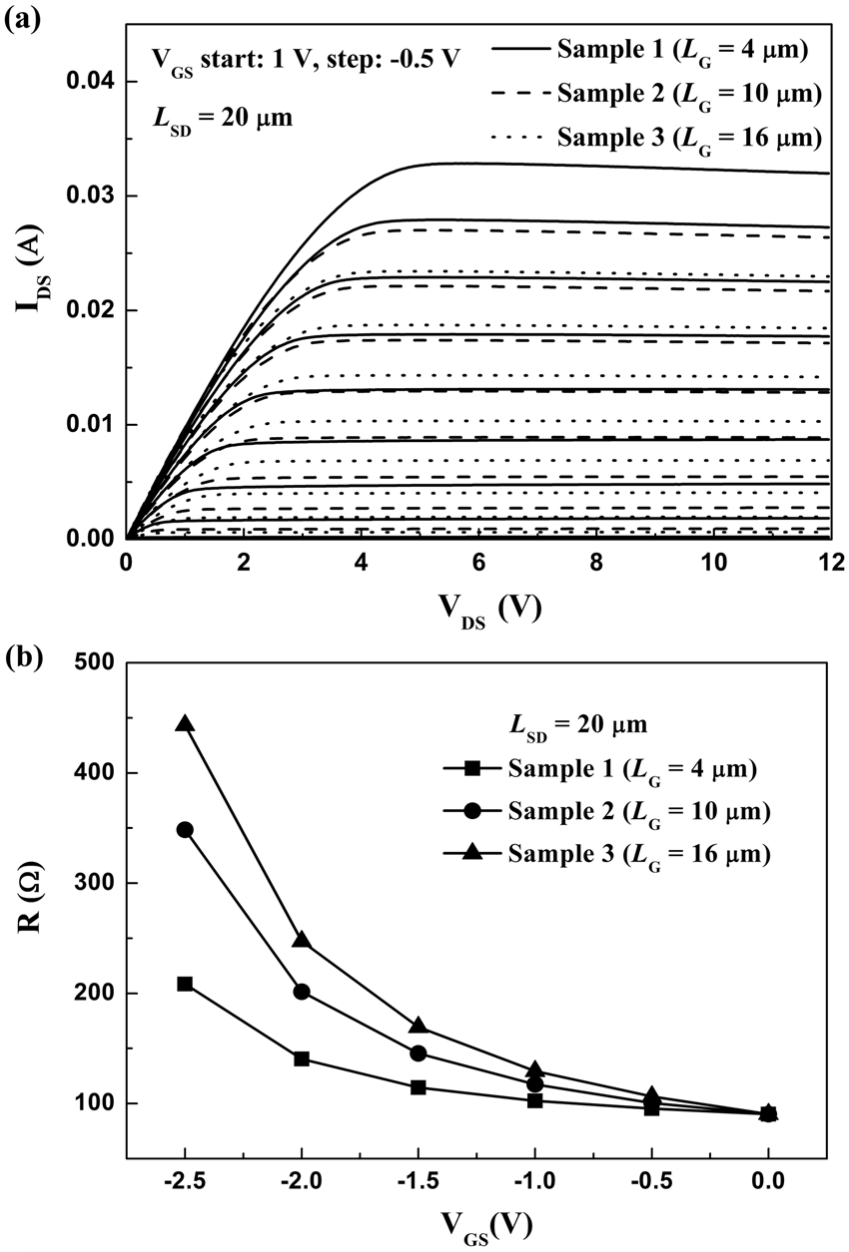
(**a**) The measured DC *I*-*V* characteristics and (**b**) the measured total resistance *R* in the source-drain channel as a function of gate bias for the three samples.

**Figure 3:**
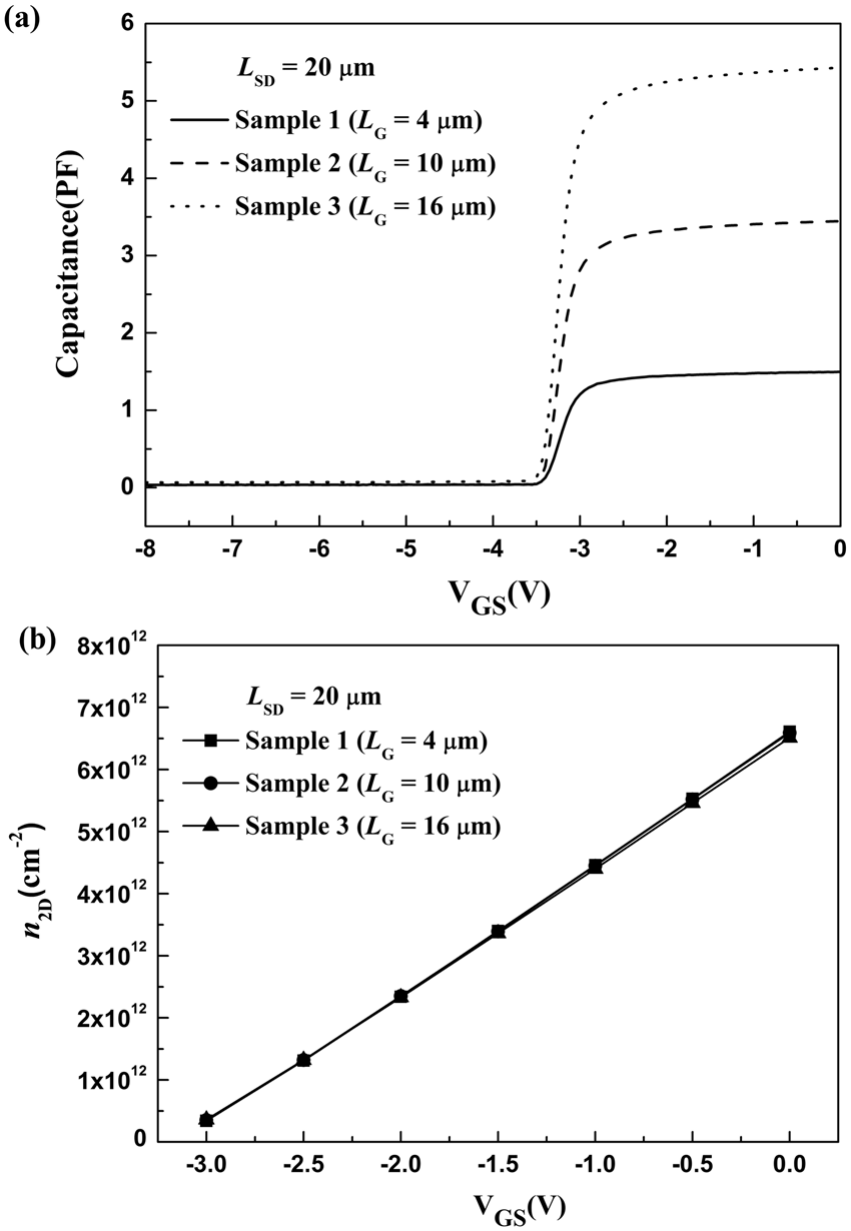
(**a**) The measured *C*-*V* characteristics and (**b**) the two-dimensional electron gas (2DEG) electron density *n*_2D_ under the gate region as a function of gate bias for the three samples.

As shown in Fig. 1, the AlGaN/GaN HFETs with source-drain spacing (*L*_SD_) of 20 μm and the gate width (*W*_G_) of 100 μm were fabricated. The Schottky gate was symmetrically placed in the middle between the source and drain ohmic contacts. The gate lengths (*L*_G_) of Sample 1, 2, and 3 are 4, 10, and 16 μm, respectively. The DC *I*-*V* characteristics for the three samples were measured, as shown in Fig. 2(a). From the *I*-*V* characteristics, the total resistance *R* in the source-drain channel as a function of gate bias can be obtained by *R* = *V*_DS_/*I*_DS_ − 2*R*_C_, as shown in Fig. 2(b). Here *I*_DS_ refers to the source-drain current corresponding to the source-drain voltage *V*_DS_ = 0.1 V. The *C*-*V* characteristics for the three samples were performed, as shown in Fig. 3(a). Integration of measured gate capacitance over gate bias yielded the two-dimensional electron gas (2DEG) electron density *n*_2D_ under the gate region^26–28,31^, as shown in Fig. 3(b).

In AlGaN/GaN HFETs, the main scattering mechanisms include polarization Coulomb field (PCF), polar optical phonon (POP), acoustic phonon (AP), interface roughness (IFR), and dislocation (DIS) scatterings^26,31,42–50^. *R* can be determined by the scattering theories of the 2DEG electrons in AlGaN/GaN HFETs^31,38,46–50^.

**Figure 4:**
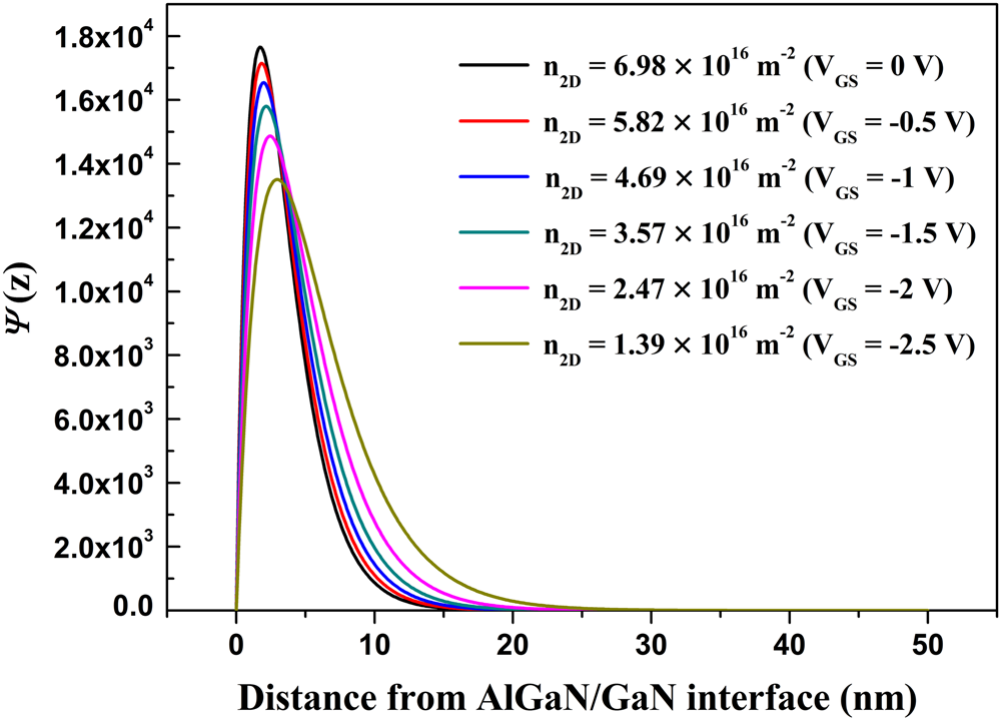
The obtained *ψ*(*z*) as a function of the distance from AlGaN/GaN interface under different *n*_2D_ (here *n*_2D_ corresponds to the electron density under the gate region as a function of gate bias).

In AlGaN/GaN HFETs, the 2DEG electrons can be written as Ψ(*x*, *y*, *z*) = *A*^−1/2^*ψ*(*z*)exp(*ik*_*x*_*x* + *ik*_*y*_*y*)^31,38,46,48,50^. Here *A* is the normalization constant, *k*_*x*_, *k*_*y*_ refer to the components of ***k*** in the *x*-direction and *y*-direction, respectively. *ψ*(*z*) = (*b*^3^*z*^2^/2)^1/2^exp(−*bz*/2) refers to the quantized wave in *z*-direction and *b* = (33m*e^2^*n*_2D_/8*ε*_0_*ε*_s_*ћ*^2^)^1/3^ is the variational parameter. m* refers to the electron effective mass of GaN, *ε*_0_ is the dielectric permittivity, and *ε*_s_ refers to the static dielectric constant of GaN. The *ψ*(*z*) under different *n*_2D_ can be calculated, as shown in Fig. 4. It is apparent *ψ*(*z*) is closely relevant with *n*_2D_. The larger *n*_2D_ is, the closer the 2DEG electrons to the AlGaN/GaN interface is. Hence, Ψ(*x*, *y*, *z*) depends on *n*_2D_. We note that *n*_2D_ under the gate region is increased with the gate bias, while *n*_2D_ under the free-contact region (including the gate-source region and the gate-drain region) is constant. Therefore, it is different for Ψ(*x*, *y*, *z*) under the gate region and the free-contact region.

**Figure 5:**
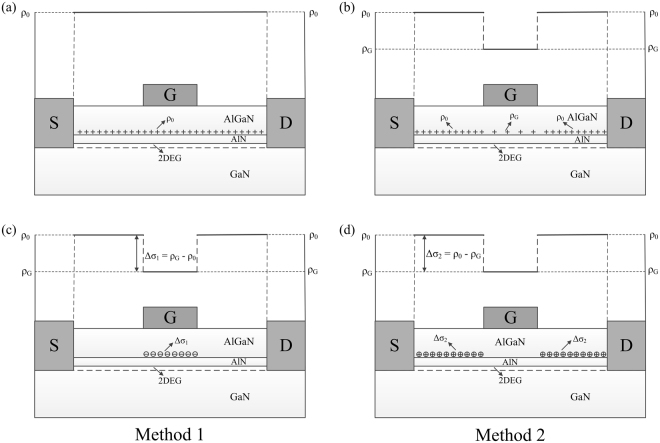
The schematic of the polarization charge distribution at the AlGaN/GaN interface (**a**) without gate bias (**b**) with the negative gate bias; The schematic of the additional polarization charge distribution at the AlGaN/GaN interface (**c**) for Method 1 and (**d**) for Method 2.

PCF scattering originates from the non-uniform distributed polarization charges at the AlGaN/GaN interface^26,27,31^. Before device processing, because of the spontaneous and piezoelectric polarization, there are uniform distributed polarization charges at the AlGaN/GaN interface, as shown in Fig. 5(a), which cannot scatter the channel electrons. The charge density of the uniform distributed polarization charges is named as ρ_0_. Due to the converse piezoelectric effect, the strain of the AlGaN barrier layer underneath the gate region can be altered by the gate bias, as shown in Fig. 5(b). The polarization charge density under the gate region, labeled as ρ_G_, can be calculated as following^36–38,41^

1 $${{\rm{\rho }}}_{{\rm{G}}}=\frac{{{e}}_{33}^{2}}{{{C}}_{{\rm{33}}}}\cdot \frac{{V}_{{\rm{GS}}}}{{d}_{{\rm{AlGaN}}}}{+{\rm{\rho }}}_{{\rm{0}}},$$

where *e*_33_ and *C*_33_ are the piezoelectric coefficient and the elastic stiffness tensor of AlGaN, respectively, *V*_GS_ is the gate-source voltage and *d*_AlGaN_ is the thickness of the AlGaN barrier layer. The free-contact region cannot be affected by the gate bias, where the polarization charges is still equal to ρ_0_. The non-uniform distributed polarization charges between the gate region and the free-contact region can induce PCF scattering potential. The difference between the non-uniform distributed polarization charges and the uniform distributed polarization charges is defined as the additional polarization charges, labeled as Δσ. The PCF scattering potential originates from Δσ, and the determination of Δσ is based on the chosen of the Hamiltonian of electrons.

The perturbation theory is used in the PCF scattering calculation. The Hamiltonian of electrons in the source-drain channel can be split into two parts $\hat{H}={\hat{H}}_{0}+\hat{H}^{\prime}$, where ${\hat{H}}_{0}$ is known as the Hamiltonian of the unperturbed system, and $\hat{H}^{\prime}$ is very small compared to ${\hat{H}}_{0}$. As a result, $\hat{H}^{\prime}$ is called the perturbation, for its effects on the energy spectrum and eigenfunctions will be small. Here $\hat{H}^{\prime}$ originates from the effect of additional polarization charges on the channel electrons, and is referred as the PCF scattering potential. When the gate bias is applied to the AlGaN barrier layer, the electrons under the gate region can be modulated. Then in the source–drain channel electron system, there are two types of ${\hat{H}}_{0}$, which are the ${\hat{H}}_{0}$ for the electrons under the gate region, and the ${\hat{H}}_{0}$ for the electrons under the free contact region. Both can likely be treated as ${\hat{H}}_{0}$.

If the ${\hat{H}}_{0}$ for the electrons under the free-contact region is chosen, as shown in Fig. 5(c), Ψ(*x*, *y*, *z*) in the free-contact region is used. This is defined as the Method 1. Because the 2DEG electron density under the free-contact region is equal to *n*_2D0_ (here *n*_2D0_ refers to the 2DEG electron density under the gate region corresponding to *V*_GS_ = 0 V), *n*_2D_ = *n*_2D0_ is used in Ψ(*x*, *y*, *z*). The polarization charges under the free-contact region is ρ_0_, therefore the additional charges can be obtained by^36–38,41^

2 $${\rm{\Delta }}{\sigma }_{1}={{\rm{\rho }}}_{{\rm{G}}}-{{\rm{\rho }}}_{0}=\frac{{e}_{33}^{2}}{{C}_{33}}\cdot \frac{{V}_{{\rm{GS}}}}{{d}_{{\rm{AlGaN}}}}.$$

The additional polarization charges Δσ_1_ is under the gate region. Based on the obtained Δσ_1_, the PCF scattering potential can be written as^31,37,38,50^

3 $${V}_{1}(x,\,y,\,z)=-\,\frac{e}{4\pi {\varepsilon }_{s}{\varepsilon }_{0}}{\int }_{-\frac{{L}_{{\rm{G}}}}{2}}^{\frac{{L}_{{\rm{G}}}}{2}}dx^{\prime} {\int }_{0}^{{W}_{G}}\frac{{\rm{\Delta }}{\sigma }_{1}}{\sqrt{{(x-x^{\prime} )}^{2}+{(y-y^{\prime} )}^{2}+{z}^{2}}}dy^{\prime} .$$

If the ${\hat{H}}_{0}$ for the electrons under the gate region is chosen, as shown in Fig. 5(d), Ψ(*x, y, z*) under the gate region is used. This is defined as the Method 2. Here *n*_2D_ under the gate region is used for Ψ(*x*, *y*, *z*). The additional polarization charges can be determined by

4 $${\rm{\Delta }}{\sigma }_{2}={{\rm{\rho }}}_{0}-{{\rm{\rho }}}_{{\rm{G}}}=-\,\frac{{e}_{33}^{2}}{{C}_{33}}\cdot \frac{{V}_{{\rm{GS}}}}{{d}_{{\rm{AlGaN}}}}\cdot $$

The additional polarization charges Δσ_2_ is under the gate-source region and the gate-drain region. Based on the obtained Δσ_2_, the PCF scattering potential can be expressed by^31,37,38,50^

5 $$\begin{array}{ccc}{V}_{2}(x,\,y,\,z) & = & -\frac{e}{4\pi {\varepsilon }_{s}{\varepsilon }_{0}}{\int }_{-{L}_{{\rm{G}}{\rm{S}}}-\frac{{L}_{{\rm{G}}}}{2}}^{-\frac{{L}_{{\rm{G}}}}{2}}dx^{\prime} {\int }_{0}^{{W}_{G}}\frac{{\rm{\Delta }}{\sigma }_{2}}{\sqrt{{(x-x^{\prime} )}^{2}+(y-y^{\prime} {)}^{2}+{z}^{2}}}dy^{\prime} \\  &  & -\,\frac{e}{4\pi {\varepsilon }_{s}{\varepsilon }_{0}}{\int }_{\frac{{L}_{{\rm{G}}}}{2}}^{{L}_{{\rm{G}}{\rm{D}}}+\frac{{L}_{{\rm{G}}}}{2}}dx^{\prime} {\int }_{0}^{{W}_{G}}\frac{{\rm{\Delta }}{\sigma }_{2}}{\sqrt{{(x-x^{\prime} )}^{2}+(y-y^{\prime} {)}^{2}+{z}^{2}}}dy^{\prime} \end{array}.$$

Here *L*_GS_ and *L*_GD_ refer to the gate-source spacing and gate-drain spacing, respectively.

In the next calculation processing, as discussed above, the different wave functions Ψ(*x*, *y*, *z*) and the PCF scattering potential *V*(*x*, *y*, *z*) are adopted for two different methods. With the obtained Ψ(*x*, *y*, *z*) and *V*(*x*, *y*, *z*), the scattering matrix element is given by^31,37,38,50^

6 $$\begin{array}{ccc}{M}_{k\to k^{\prime} } & = & {A}^{-1}{\int }_{0}^{{\rm{\infty }}}{\psi }_{k^{\prime} }^{\ast }(z)[{\int }_{-\frac{{L}_{{\rm{S}}{\rm{D}}}}{2}}^{\frac{{L}_{{\rm{S}}{\rm{D}}}}{2}}dx{\int }_{0}^{{W}_{G}}V(x,\,y,\,z)\\  &  & \times \,\exp (\,-\,i{q}_{x}x-i{q}_{y}y)\,dy]{\psi }_{k}(z)dz\\  & = & {A}^{-1}{\int }_{0}^{{\rm{\infty }}}{\psi }_{k^{\prime} }^{\ast }(z)[V({q}_{x},\,{q}_{y},\,z)]{\psi }_{k}(z)dz.\end{array}$$

where *q*_*x*_, *q*_*y*_ refer to the components of the wave vector ***q*** in the *x*-direction and *y*-direction, respectively. The change in ***q*** during a scattering process is related to the scattering angle *θ* between initial state ***k*** and final state ***k***′ by *q* = |2(2m*ℏ^−2^*E*) ^1/2^sin (*θ*/2)|.

Then the energy dependent scattering rate for the PCF scattering can be obtained^31,37,38,50^

7 $$\frac{1}{{\tau }_{{\rm{PCF}}}(E)}=\frac{A{m}^{\ast }}{2\pi {\hslash }^{3}}{\int }_{-\pi }^{\pi }{|\frac{{M}_{k\to k^{\prime} }}{S(q,{T}_{e})}|}^{2}(1-\,\cos \,\theta )d\theta .$$

where *ћ* is the Planck constant. The screening function *S* (*q*, *T*_e_) is^31,37,38,50^

8 $$S(q,{T}_{e})=1+\frac{{e}^{2}F(q)\prod (q,{T}_{e},E)}{2{\varepsilon }_{0}{\varepsilon }_{{\rm{s}}}q}.$$

Here the form factor *F* (*q*) is^31,37,38,50^

9 $$F(q)={\int }_{0}^{\infty }{\int }_{0}^{\infty }{\psi }^{2}(z){\psi }^{2}(z^{\prime} )\,\exp \,(\,-\,q|z-z^{\prime} |dzdz^{\prime} ).$$

The closer the 2DEG electrons to the additional polarization charges is, the stronger the Coulomb screening effect is. Therefore, the 2DEG channel electrons, which are the nearest to the additional polarization charges, are chosen in Eq. (9). That is, for Method 1, Δσ_1_ is under the gate region and the electron wave function under the gate region is used in Eq. (9). Conversely, for Method 2, Δσ_2_ is under the free-contact region and the electron wave function under the free-contact region is used in Eq. (9).

The polarizability function Π (*q*, *T*_e_, *E*) is^31,37,38,50^

10 $$\prod (q,{T}_{e},E)=\frac{{m}^{\ast }}{4\pi {\hslash }^{2}{k}_{{\rm{B}}}{T}_{e}}{\int }_{0}^{{\rm{\infty }}}\frac{1-{\rm{\Theta }}(q-2{k}_{{\rm{F}}}){[1-{(2{k}_{{\rm{F}}}/q)}^{2}]}^{1/2}}{{\cosh }^{2}[({E}_{{\rm{F}}}-E)/2{k}_{{\rm{B}}}{T}_{e}]}dE.$$

In this equation, Θ(*x*) is the usual step function, *k*_F_ = (2*πn*_2D_)^1/2^ is the Fermi wave vector, *T*_e_ is the electron temperature, *E*_F_ is the Fermi energy, and *E* is the energy.

At the end, the momentum relaxation time *τ*_PCF_ for PCF scattering can be got^31,37,38,50^

11 $${\tau }_{{\rm{PCF}}}=\int {\tau }_{{\rm{PCF}}}(E)E\frac{\partial {f}_{0}(E)}{\partial E}dE/\int E\frac{\partial {f}_{0}(E)}{\partial E}dE.$$

where *f*_0_, the Fermi function, is

12 $${f}_{0}(E)=\frac{1}{\exp [(E-{E}_{{\rm{F}}})/{k}_{{\rm{B}}}{T}_{e}]+1}.$$

Here *k*_B_ is the Boltzmann constant.

The momentum relaxation times *τ*_POP_, *τ*_AP_, *τ*_IFR_, and *τ*_DIS_ for POP, AP, IFR, and DIS scatterings were obtained by the pre-existing calculation formulas^31,37,38,50^. Considering *n*_2D_ between the gate region and the free-contact region is different, the *R* for three samples can be determined with the resistance *R*_G_ under the gate region plus the resistance *R*_F_ under the free-contact region^31,37,38,50^.

13 $$\begin{array}{ccc}R={R}_{{\rm{G}}}+{R}_{{\rm{F}}} & = & \frac{{m}^{\ast }{L}_{G}}{{n}_{2{\rm{D}}}{e}^{2}{W}_{{\rm{G}}}}(\frac{1}{{\tau }_{{\rm{P}}{\rm{C}}{\rm{F}}}}+\frac{1}{{\tau }_{{\rm{P}}{\rm{O}}{\rm{P}}}^{{\rm{G}}}}+\frac{1}{{\tau }_{{\rm{A}}{\rm{P}}}^{{\rm{G}}}}+\frac{1}{{\tau }_{{\rm{I}}{\rm{F}}{\rm{R}}}^{{\rm{G}}}}+\frac{1}{{\tau }_{{\rm{D}}{\rm{I}}{\rm{S}}}^{{\rm{G}}}})\\  &  & +\,\frac{{m}^{\ast }({L}_{{\rm{G}}{\rm{S}}}+{L}_{{\rm{G}}{\rm{D}}})}{{n}_{2{\rm{D}}0}{e}^{2}{W}_{{\rm{G}}}}(\frac{1}{{\tau }_{{\rm{P}}{\rm{C}}{\rm{F}}}}+\frac{1}{{\tau }_{{\rm{P}}{\rm{O}}{\rm{P}}}^{{\rm{F}}}}+\frac{1}{{\tau }_{{\rm{A}}{\rm{P}}}^{{\rm{F}}}}+\frac{1}{{\tau }_{{\rm{I}}{\rm{F}}{\rm{R}}}^{{\rm{F}}}}+\frac{1}{{\tau }_{{\rm{D}}{\rm{I}}{\rm{S}}}^{{\rm{F}}}}).\end{array}$$

**Figure 6:**
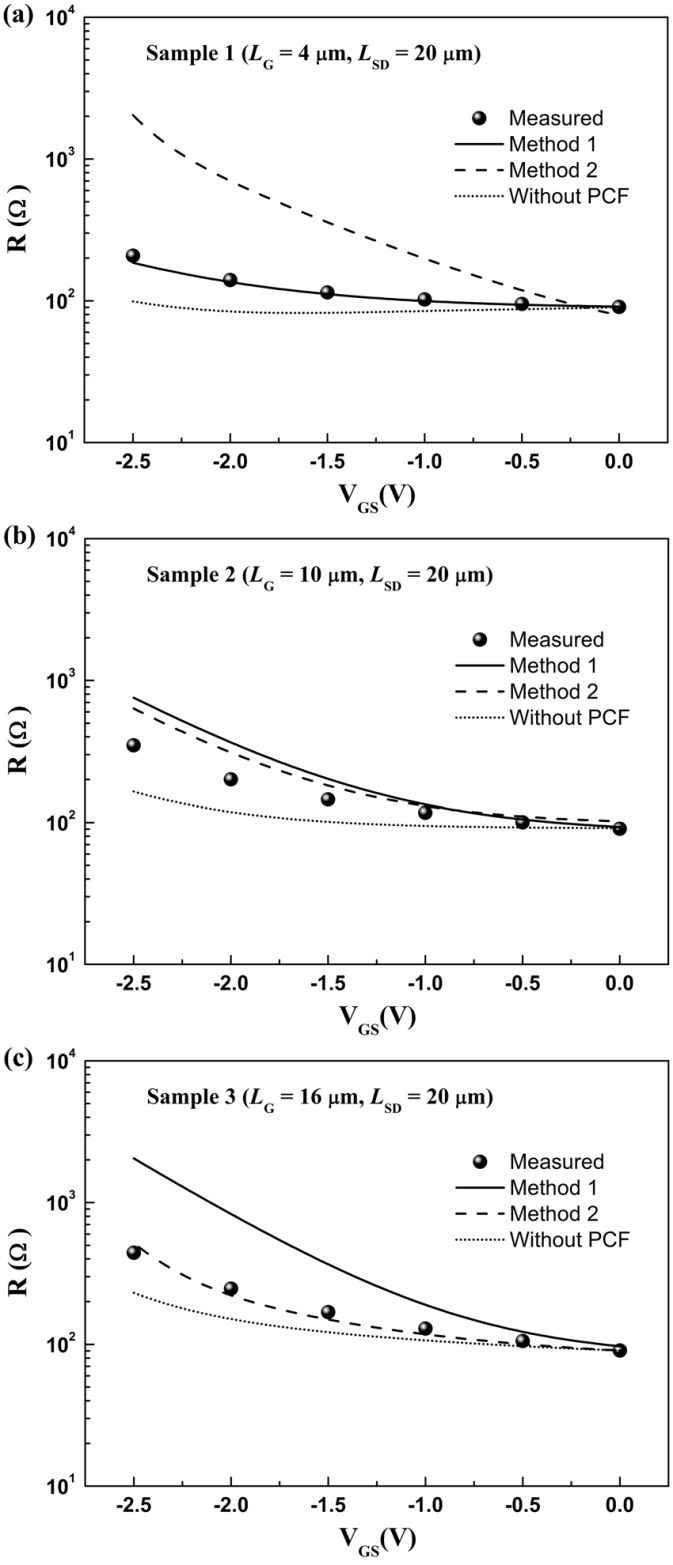
The measured source-drain channel resistance (Measured), and the calculated source-drain channel resistance calculated by Method 1 (Method 1), Method 2 (Method 2) as well as without PCF scattering (Without PCF), as a function of gate bias for (**a**) Sample 1, (**b**) Sample 2, and (**c**) Sample 3, respectively.

Here ${\tau }_{{\rm{POP}}}^{{\rm{G}}}$, ${\tau }_{{\rm{AP}}}^{{\rm{G}}}$, ${\tau }_{{\rm{IFR}}}^{{\rm{G}}}$, and ${\tau }_{{\rm{DIS}}}^{{\rm{G}}}$ refer to the momentum relaxation times for POP, AP, IFR, and DIS scatterings under the gate region, respectively. ${\tau }_{{\rm{POP}}}^{{\rm{F}}}$, ${\tau }_{{\rm{AP}}}^{{\rm{F}}}$, ${\tau }_{{\rm{IFR}}}^{{\rm{F}}}$, and ${\tau }_{{\rm{DIS}}}^{{\rm{F}}}$ refer to the momentum relaxation times for POP, AP, IFR, and DIS scatterings under the free-contact region, respectively. The calculated *R* for the three samples was shown in Fig. 6. Firstly, there is a distinct difference between the measured *R* and the calculated *R* excluding PCF scattering. This means PCF scattering is not ignorable in AlGaN/GaN HFETs. Then *R*, including the calculated PCF scattering by the two methods, was obtained. It is apparent that the calculated results by Method 1 has better accord with the measured values for Sample 1, and the calculated results by Method 2 agree well with the measured values for Sample 3. Different from the two samples, the calculated results by both Method 1 and Method 2 have a significant difference with the measured values for Sample 2. This phenomenon can be explained as following.

POP, AP, IFR, and DIS scatterings are consistent for the three samples, therefore the difference should come from the PCF scattering calculation with the two methods. PCF scattering is calculated by the scattering model based on the perturbation theory. The perturbation theory is most suitable when $\hat{H}$ is very close to ${\hat{H}}_{0}$ and PCF scattering can be solved exactly. To this end, H′ should be far less than H_0_. The less H′ is, the more precise the calculated PCF scattering is. H′ is the PCF scattering potential *V* (*x*, *y*, *z*). Therefore, for a more clear presentation, the average value of *V* (*x*, *y*, *z*) can be expressed by^36–38^

14 $$\bar{V}=\frac{1}{{L}_{SD}\cdot {W}_{G}\cdot z}{\int }_{-\frac{{L}_{{\rm{SD}}}}{2}}^{\frac{{L}_{{\rm{SD}}}}{2}}dx^{\prime} {\int }_{0}^{{W}_{G}}dy{\int }_{0}^{z}V(x,y,z)dz.$$

**Figure 7:**
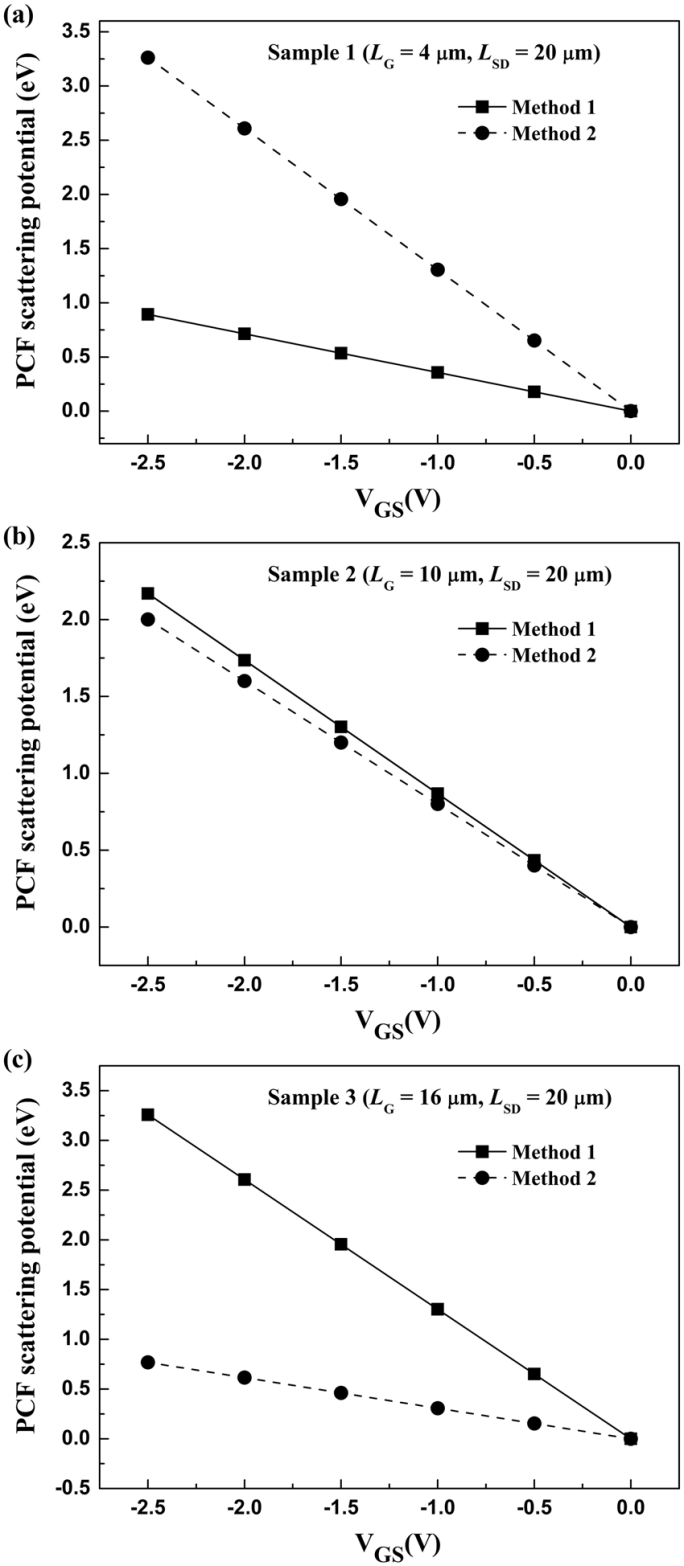
The obtained average value of *V* (*x*, *y*, *z*) by two methods as a function of gate bias for (**a**) Sample 1, (**b**) Sample 2, and (**c**) Sample 3, respectively.

Here, considering the 2DEG wave function distribution in *z*-direction, as shown in Fig. 4, *z* = 50 nm is adopted. The calculated $\overline{V}$ with two methods for the three samples was shown in Fig. 7.

For sample 1, because *L*_G_ = 4 μm and *L*_SD_ = 20 μm, the free-contact region is larger than the gate region. With Method 2, Δσ_2_ and *V*_2_ (*x*, *y*, *z*) are used. Because of the large free-contact region, there are numerous additional polarization charges under the free-contact region, which will induce a large PCF scattering potential, namely a larger H′. The large H′ can affect the precision of the calculated PCF scattering. Conversely, with Method 1, Δσ_1_ and *V*_1_ (*x*, *y*, *z*) are adopted. Due to the small gate length, the additional polarization charges underneath the gate region is small for a large source-drain distance, leading to smaller *V* (*x*, *y*, *z*) or H′. As shown in Fig. 7(a), the $\overline{V}$ obtained by Method 1 is smaller than that obtained by Method 2. The smaller $\overline{V}$ means a less H′, which can improve the precision of the PCF scattering calculation. This means for a small gate length, it is appropriate that *V*_1_ (*x*, *y*, *z*) is treated as the PCF scattering potential.

For Sample 3, because *L*_G_ = 16 μm and *L*_SD_ = 20 μm, the gate region is larger than the free-contact region. As discussed above, in order to obtain a small H′, the additional polarization charges under the free-contact region should be chosen. This means Δσ_2_ and *V*_2_ (*x*, *y*, *z*) should be adopted. As shown in Fig. 7(c), the $\overline{V}$ obtained by Method 2 is smaller than that obtained by Method 1. Hence Method 2 is more suitable for the PCF scattering calculation, which agrees well with the results as shown in Fig. 6(c). This indicates that for a large gate length, *V*_2_ (*x*, *y*,* z*) should be adopted as the PCF scattering potential.

For Sample 2, on account of *L*_G_ = 10 μm and *L*_SD_ = 20 μm, the gate region is equal to the free-contact region. H′ obtained by the two methods both cannot be small enough, as shown in Fig. 7(b), which affect the precision of the PCF scattering calculation. Therefore, the obtained *R* by two methods has a significant difference with the measured values.

In addition, as the gate bias is more negative, the number of additional polarization charges (Δσ_1_ and Δσ_2_) is increased. The increased Δσ can increase the *V* (*x*, *y*, *z*) and H′, as show in Fig. 7, which also can reduce the precision of the PCF scattering calculation. That is why as the gate bias is decreased, the deviation between the calculated *R* and the measured *R* is increased. This further indicated that the explanation for the effect of different gate lengths on PCF scattering potential is suitable.

## Conclusions

In summary, with the measured and calculated source-drain channel resistances for the AlGaN/GaN HFETs with different gate lengths, the effect of different gate lengths on PCF scattering potential was analyzed. It is found that the gate length can influence the determination of the Hamiltonian of the system and the additional polarization charges, and then affect the PCF scattering potential. This study offers an effective way for improving the precision of the PCF scattering model.

## Methods

### Sample fabrication

The AlGaN/GaN heterostructure was grown by molecular beam epitaxy (MBE) on a sapphire substrate. The epitaxial structure consists of, from the bottom to the top, a 100-nm-thick AlN nucleation layer, a 1-μm-thick GaN buffer layer with carbon doped, a 1-μm-thick GaN channel layer, a 0.7-nm-thick AlN interlayer, a 25.5-nm-thick AlGaN barrier layer with 21% Al composition, and a 3-nm-thick GaN cap layer. A high sheet electron density (*n*_2D_) of 8.4 × 10^12^ cm^−2^ and a high electron mobility (*μ*) of 2340 cm^2^/V∙s were obtained by Hall measurement. Device isolation was achieved by inductively coupled plasma reactive ion etching (ICP-RIE) using BCl_3_/Cl_2_ gas mixture. Ti/Al/Ni/Au-based ohmic metal stack was then deposited and annealed in N_2_ ambient at 850 °C for 30 s. The ohmic contact resistance *R*_C_ = 10 Ω was obtained by the transmission line method (TLM). Finally, a Ni/Au metal stack was deposited to form the gate electrode. The source-drain spacing (*L*_SD_) is 20 μm, and the gate width (*W*_G_) is 100 μm. The Schottky gate was symmetrically placed in the middle between the source and drain ohmic contacts. As shown in Fig. 1(b), the gate lengths (*L*_G_) of Sample 1, 2, and 3 were 4 μm, 10 μm and 16 μm, respectively.

### Measurements

Current-voltage (*I*-*V*) measurements and capacitance-voltage (*C*-*V*) measurements were performed by using an Agilent B1500A and Agilent B1520A Semiconductor Parameter Analyzers, respectively.
